# The Role of Epicardial Adipose Tissue in the Development of Atrial Fibrillation: A Systematic Review and Meta-Analysis

**DOI:** 10.14740/jocmr6465

**Published:** 2026-02-28

**Authors:** Aida I. Tarzimanova, Anna E. Bragina, Liubov A. Ponomareva, Liubov V. Vasileva, Daria D. Vanina, Ilya I. Shvedov, Anna E. Pokrovskaya, Tatiana A. Safronova, Tatiana S. Vargina, Irakli Zh. Loriya, Elena N. Popova, Paria Shooriberis, Yaroslav M. Malinin, Valery I. Podzolkov

**Affiliations:** a Department of Internal Medicine No. 2, Institute of Clinical Medicine, Sechenov University, Moscow, Russia; b Synergy University, Moscow, Russia; c Tareev Clinic of Internal Diseases, Sechenov University, Moscow, Russia

**Keywords:** Atrial fibrillation, Total epicardial adipose tissue, Periatrial epicardial adipose tissue

## Abstract

**Background:**

Atrial fibrillation (AF) is the most frequent arrhythmia worldwide that significantly elevates stroke and heart failure risks. Recent developments in imaging research have shown the need for exploring epicardial adipose tissue (EAT) as a contributor to atrial pathology.

**Methods:**

Following the Preferred Reporting Items for Systematic Reviews and Meta-Analyses (PRISMA) guidelines and registered in PROSPERO (CRD42022360443), a systematic search was conducted across PubMed, Scopus and Google Scholar using terms related to AF and EAT quantified using computed tomography. Inclusion criteria encompassed *in vivo* studies assessing EAT’s effect on AF, with reported outcomes including AF development. Publication bias was assessed through two complementary approaches: visual inspection of funnel plot symmetry and formal statistical testing using Egger’s or Begg’s tests. A two-tailed P value threshold of 0.05 was established for determining statistical significance throughout all analyses.

**Results:**

Ten studies (851 patients) analyzed showed the relationship between total EAT and AF. Meta-analysis of aggregate data revealed a statistically significant standardized mean difference (SMD) of 0.70 (95% confidence interval (CI), 0.24–1.15; I^2^ = 91%; P < 0.01). Seven studies (579 patients) analyzed the relationship between periatrial EAT and AF. Meta-analysis of aggregate data revealed a statistically significant SMD of 1.13 (95% CI, 0.49–1.78; I^2^ = 91%; P < 0.01).

**Conclusions:**

This meta-analysis demonstrates that total and periatrial EAT correlate with AF; however, periatrial EAT has a more convincing association with AF than total EAT.

## Introduction

AF is the most common heart arrhythmia worldwide, significantly increasing the risk of stroke, heart failure, and cardiovascular mortality. With its rising prevalence and the limitations of current therapies, identifying novel risk factors is essential for advancing patient care. Epicardial adipose tissue (EAT) has emerged as promising target, potentially revolutionizing AF prediction, prevention, and management through its inflammatory and metabolic roles.

EAT is a critical contributor to AF pathogenesis [1]. Studies consistently link greater EAT volume or thickness to increased AF incidence, severity, and recurrence, independent of generalized obesity [2, 3]. For instance, Abe et al showed that EAT-derived inflammatory cytokines, such as IL-G and YKL-40, promote atrial fibrosis, a key substrate for AF [4]. This association is evident in clinical contexts like catheter ablation or cardiac surgery, where EAT predicts post-procedural AF recurrence [5, 6]. Recent research highlights EAT’s qualitative traits linking higher EAT radiodensity to electrophysiological changes, such as prolonged P-wave dispersion and AF recurrence after ablation [7, 8]. Meta- analyses confirm EAT volume as a robust predictor of AF relapse [9, 10]. As a mediator in obesity- and diabetes-related AF, EAT bridges metabolic disorders to cardiac arrhythmia, emphasizing its clinical relevance amid the global obesity epidemic [11, 12]. These findings position EAT as a potential biomarker and therapeutic target for AF management.

Considering the contribution of EAT to AF, this systematic review and meta-analysis, covering studies from 2015 to 2025, aims to comprehensively evaluate the relationship between total or periatrial EAT and AF based on recent investigations.

## Materials and Methods

This systematic review and meta-analysis was performed in accordance with the Preferred Reporting Items for Systematic Reviews and Meta-Analyses (PRISMA) guidelines [13]. The predefined protocol was registered in PROSPERO database (CRD42022360443). An amendment to the original protocol was made on January 15, 2026, refining the primary objective from “To assess the importance of epicardial adipose tissue in the occurrence of new cases and frequency of atrial fibrillation (AF) paroxysms and the effect on progression of AF from paroxysmal to permanent form” to “To assess the importance of epicardial adipose tissue in the occurrence of new cases and frequency of AF paroxysms,” due to insufficiency of available literature to perform a robust meta-analysis of this pre-specified outcome.

The Institutional Review Board approval was not required since this study is a systematic review and meta-analysis. The study was conducted in compliance with the ethical standards of the responsible institution on human subjects as well as with the Helsinki Declaration.

### Search strategy

The information search algorithm was developed according to the reporting requirements and provisions for systematic reviews and meta-analyses (PRISMA) in the PubMed, Scopus, Google Scholar databases and included a search for studies using following keywords: (epicardial fat OR pericardial fat OR epicardial adipose tissue OR pericardial adipose tissue) AND (atrial fibrillation development OR atrial fibrillation progression OR atrial fibrillation occurrence OR atrial fibrillation incidence) NOT fat pad removal. A systematic literature search was conducted from 2015 to 2025. Publications not in English or Russian were excluded.

### Selection criteria

Two independent reviewers (LP and DV) screened all articles retrieved during the initial search for title and abstract compliance. Studies potentially meeting the selection criteria were further reviewed for inclusion in the final analysis based on the full text. Only studies that adequately reported the original data—assessment of “parameter that was assessed and by what it was assessed, category of patients”—were included in the systematic review. An obligatory criterion for inclusion in the meta-analysis was the availability of data presented with an indication of the mean and standard deviation (SD). The authors independently examined the titles and abstracts of publications for compliance with the inclusion criteria. Any disagreements were resolved through negotiations. Publications in which quantitative data were presented as median and interquartile range (Q1; Q3)) were excluded from the analysis. In addition, we removed duplicates, reviews, reports, books, clinical cases, and studies in which other instrumental methods were used, and animal studies.

### Data extraction

Data of interest were extracted by two independent reviewers (LP and DV) and crosschecked for any disagreements. Author, country of publication, study size, epicardial fat measurement method, and measurement results were extracted. Baseline characteristics of each study population were also documented.

### Study quality assessment

The quality of observational studies was evaluated by the Newcastle-Ottawa Scale (NOS) quality assessment. The assessment elements included the study group’s selection, comparability of groups, and ascertainment of the exposure and outcomes. Seven scores or more was regarded as high-quality study. Leave-one-out sensitivity analysis was performed to assess the influence of individual studies. The results remained consistent regardless of which study was removed.

### Statistical analyses

The statistical analyses were conducted using R software (version 4.3) with specialized packages including “meta”, “metafor”, and “dmetar.” For each study, standardized mean difference (SMD) and its 95% confidence interval (CI) were calculated based on the means and SDs of EAT volume in groups of patients with AF versus controls. Effect sizes were expressed as Hedges’ g to account for small sample biases. For the meta-analysis, we computed pooled SMD through a random effects model, analyzing total EAT and periatrial EAT measurements separately. The between-study variance (τ^2^) was estimated using the restricted maximum-likelihood (REML) approach.

To evaluate heterogeneity across studies, we used Cochran’s Q test and calculated the I^2^ statistic according to Higgins and Thompson methods. We performed leave-one-out sensitivity analyses to examine the impact of each individual study on the overall results. For continuous variables such as publication year, participant age, and proportion of male subjects, meta-regression analyses were conducted. Given the limited number of studies, a P value < 0.1 was considered indicative of a potential moderating effect in exploratory meta-regression analyses aimed at identifying sources of heterogeneity.

Publication bias was assessed through two complementary approaches: visual inspection of funnel plot symmetry and formal statistical testing. Specifically, Egger’s regression method was used for analyses including 10 or more studies, while Begg’s rank correlation test was applied to those with fewer than 10 studies. A two-tailed P value threshold of 0.05 was established for determining statistical significance for the primary meta-analyses. Level of significance of P < 0.10 was used in the meta-regression analyses due to small number of studies.

## Results

### Search results and studies characteristics

**Figure 1:**
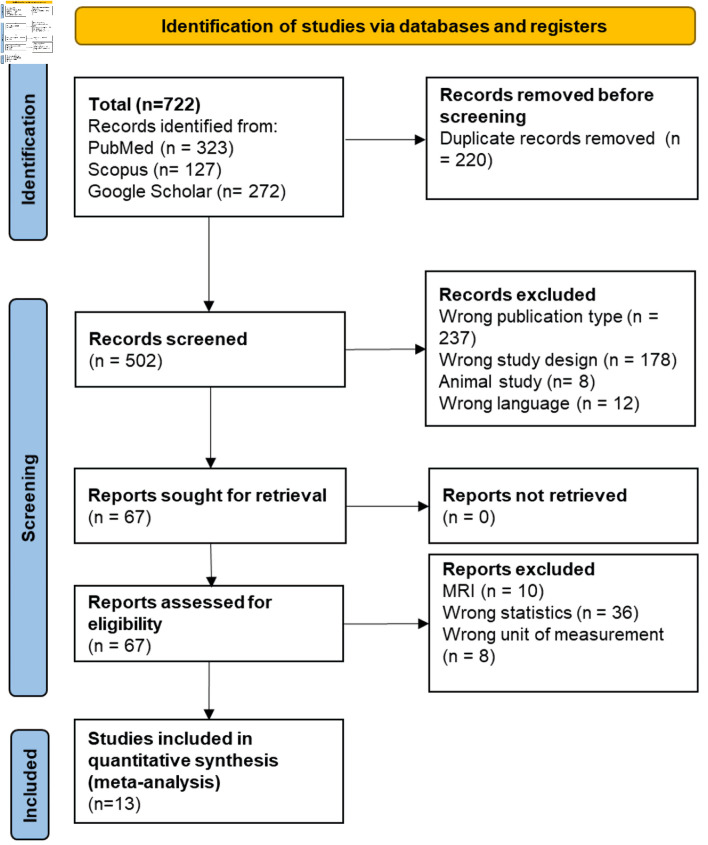
Flowchart of selection of studies for inclusion in the meta-analysis. MRI: magnetic resonance imaging.

The flow of study search and selection are depicted in Figure 1. A total of 722 articles were retrieved by electronic search. Thirteen articles were considered to be eligible and were included in the final analysis [14–26]. By full-text examination, 13 articles were eventually included for data synthesis with 2,117 participants. In all selected studies the patients had idiopathic paroxysmal or persistent form of AF. Quantification of EAT was carried out by computed tomography (CT) (ranges from –50 to –200 Hounsfield units (HU)). In five studies we analyzed the role of EAT after surgical treatment of AF [16, 17, 20, 23, 24]. General study characteristics are shown in Table 1 [14–26].

**Table 1:** Characteristics of the Included Studies

Author (year)	Country	Study design	N	N with AF	Mean age (years)	Male (%)	BMI (kg/m^2^)	Method	Object
Tsao et al. 2016 [14]	Republic of China	Cross-sectional	88	68	63.4	76.5	25.2	CT	Periatrial
Oba et al. 2018 [15]	Japan	Cross-sectional	316	204	64.0	72.0	25.8	CT	Total
Maeda et al. 2018 [16]	Japan	Cross-sectional	218	61	64.9	67.2	26.0	CT	Total
Monno et al. 2018 [17]	Japan	Cross-sectional	104	23	62.0	65.0	24.1	CT	Total and periatrial
Ozbek et al. 2018 [18]	Turkey	Cross-sectional	149	35	69.2	65.7	29.3	CT	Total
Nikitin et al, 2021 [19]	Russia	Cross-sectional	60	45	55.2	55.5	31.3	CT	Total and periatrial
Yang et al, 2022 [20]	China	Cross-sectional	348	174	64.0	63.8	24.6	CT	Total and periatrial
Sun et al, 2022 [21]	China	Cross-sectional	49	24	60.4	58.3	25.7	CT	Periatrial
Shao et al, 2022 [22]	China	Cross-sectional	276	214	62.0	66.8	25.1	CT	Periatrial
Jian et al, 2022 [23]	China	Cross-sectional	337	235	55.1	53.0	25.3	CT	Total
Ilyushenkova et al, 2022 [24]	Russia	Cross-sectional	43	19	43.8	89.4	27.9	CT	Total
Marinelli et al, 2023 [25]	Italy	Cross-sectional	62	31	27.9	90.3	24.3	CT	Total and periatrial
Deng et al, 2023 [26]	China	Cross-sectional	67	24	61.6	79.2	25.7	CT	Total

BMI: body mass index; AF: atrial fibrillation; CT: computed tomography.

### Relationship between total EAT and AF

**Figure 2:**
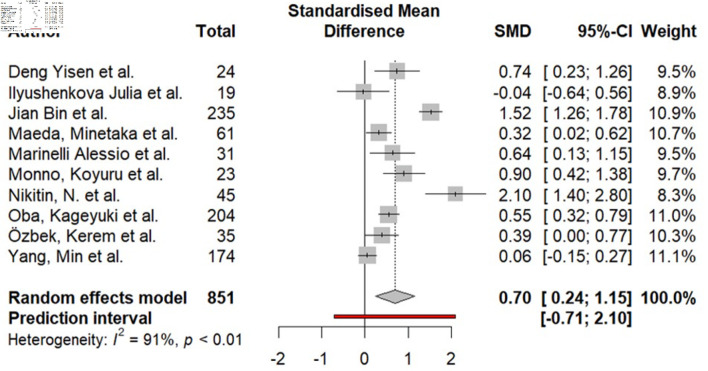
Difference in total epicardial fat (in mL) in the group of patients with AF compared to the group without AF. AF: atrial fibrillation; SMD: standardized mean difference; CI: confidence interval.

Ten studies (851 patients) analyzed showed the relationship between total EAT and AF (Fig. 2). Meta-analysis of aggregate data revealed a statistically significant SMD of 0.70 (95% CI, 0.24–1.15; I^2^ = 91%; P < 0.01).

**Figure 3:**
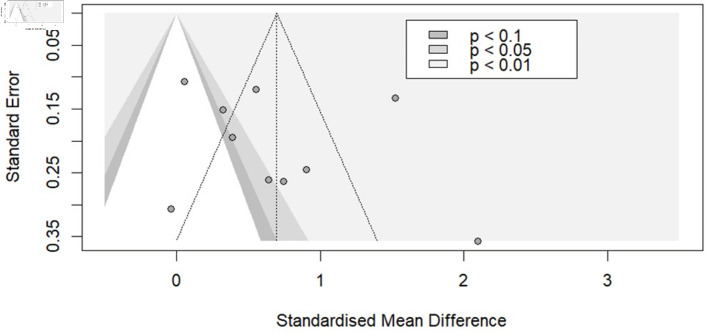
Funnel plot for studies that evaluated the relationship between total EAT and AF. EAT: epicardial adipose tissue; AF: atrial fibrillation.

A funnel plot and Egger’s test (P = 0.470) were used to assess potential publication bias and demonstrated no publication bias (Fig. 3).

No significant confounding effect of age and publication year with AF was revealed in the meta-regression analysis. However, meta-regression indicated that the heterogeneity of the obtained values of the SMD of total EAT in the groups with and without AF may be explained by differences in the gender composition of the studies, though the significance of this finding was weak (P = 0.095) and could be subject to type I error.

### Relationship between periatrial EAT and AF

**Figure 4:**
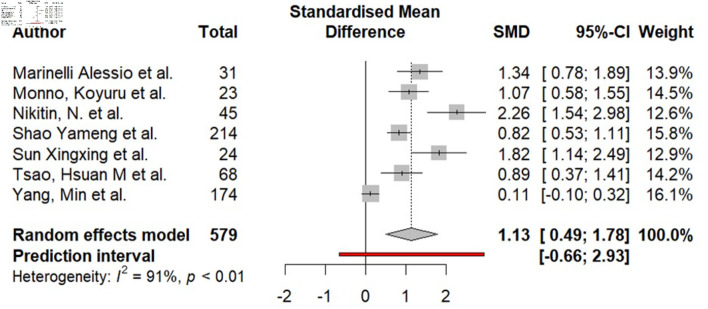
Difference in periatrial epicardial fat (in mL) in the group of patients with AF compared to the group without AF. AF: atrial fibrillation; SMD: standardized mean difference; CI: confidence interval.

Seven studies (579 patients) analyzed showed the relationship between periatrial EAT and AF (Fig. 4). Meta-analysis of aggregate data revealed a statistically significant SMD of 1.13 (95% CI, 0.49–1.78; I^2^ = 91%; P < 0.01).

Begg’s test showed statistically significant evidence of funnel plot asymmetry (P = 0.010), suggesting potential small-study effects and the possible presence of publication bias. No significant confounding effect of age, publication year and gender with AF was revealed in the meta-regression analysis. However, the differences in mean BMI were significant in the conducted meta-regression (P = 0.086). Thus, the high heterogeneity of the obtained values of the SMD of periatrial EAT in the groups with and without AF may be explained by differences in BMI.

## Discussion

This meta-analysis demonstrates that total and periatrial EAT correlate with AF. Periatrial EAT has a more convincing association with AF than total EAT, with SMD of 1.13 (95% CI, 0.49–1.78; P < 0.01). This finding, while preliminary, suggests that periatrial EAT’s proximity to the atria amplifies its impact on AF pathogenesis. Contrary to expectations, there was no significant difference between the total EAT volume in AF patients and controls beyond a moderate effect, with SMD of 0.70 (95% CI, 0.24–1.15; P < 0.01). This finding suggests that total EAT’s role in AF may be influenced by study-specific factors like imaging method or population characteristics from 2015 to 2025. Although both periatrial and total EAT demonstrate correlation with AF, these relationships are rather controversial due to the high heterogeneity of the results which could not always be explained, so future studies are necessary.

Results of previous meta-analyses in this topic were equivocal, especially regarding the role of total EAT. Several meta-analyses did not prove a significant association between total EAT and AF [10, 27, 28]. On the other hand, there are studies that showed an important role of both total and periatrial EAT in the development of AF [29, 30]. Our results show that periatrial EAT is more important than total EAT in the AF development.

There are several mechanisms of action that can explain the role of EAT in the AF development. Since there is no fascial layer between the EAT and the myocardium, epicardial fat directly affects the underlying cardiomyocytes. Infiltration of atrial myocardium by adipocytes causes circulatory disorders, hypoxia and, consequently, hypokinesia of the left atrium. Adipocytokines secreted by EAT also have anticontractive properties. The resulting dilation of the left atrium is a recognized trigger of AF. It is reported that an increased volume of EAT is associated with an increase in the diameter of the left atrium, independent of other AF risk factors [14, 15].

Fatty infiltration due to excess EAT separates the myocytes in the atrial myocardium, disrupting intercellular communication between cardiomyocytes, which directly leads to impaired conduction, the appearance of multiple, chaotic pathways of excitation, and the facilitation of the reentry mechanism. The resulting electrical remodeling of the myocardium is aggravated by changes in the activity of the cardiac autonomic nervous system in patients with excessive amounts of EAT. There is a large number of vegetative ganglia located in the EAT and around the mouths of the pulmonary veins. Adipocytokine secretion leads to increased adrenergic activation of the ganglion plexuses and increased catecholamine levels in patients with excess epicardial fat, which provoke the development and persistence of AF [1, 19, 26].

Nowadays, it is well established that EAT acts as an endocrine organ promoting AF via inflammation and oxidative stress [31]. For instance, markers of inflammation that are secreted by epicardial fat, such as C-reactive protein, interleukin (IL)-6, IL-8, IL-1b, and tumor necrosis factor (TNF)-α, have been associated with the incidence, severity, and recurrence of AF. These markers may have local pro-inflammatory effects on the adjacent atrial myocardium that facilitate arrhythmogenesis [32]. Indeed, a number of researchers showed that the concentration of inflammatory cells and inflammatory mediators in the periatrial EAT in patients with AF is higher than EAT surrounding other cardiac chambers [20, 23, 32].

Adipocytokines secreted by EAT also have a proarrhythmogenic effect due to activation of inflammation. In patients with AF, the secretion of pro-inflammatory adipocytokines (visfatin, resistin, leptin) in EAT prevails over anti-inflammatory ones (adiponectin, apelin). Visfatin, for example, can enhance lipolysis, causing increased release of free fatty acids, activating the processes of atherogenesis and lipotoxic damage to the myocardium and eventually fibrosis. Yang et al demonstrated that visfatin was an independent risk of AF recurrence post radiofrequency ablation [20, 23].

Thus, an excessive amount of EAT is associated with the development of atrial fibrosis, which is the core of the AF maintenance mechanism. Wang et al (2018) showed that connective tissue growth factor (cTGF) expression was significantly higher in EAT from patients with AF than in EAT from patients with sinus rhythm (SR) (AF EAT vs. SR EAT, 10.23 ± 5.69 vs. 2.39 ± 1.38, P < 0.001), supporting the mechanism of fibrotic atrial remodeling [33]. The concentration of matrix metalloproteinases that increase the activity of atrial myocardial fibrogenesis is also increased in EAT in patients with AF [22]. We can suggest that total EAT reflects a systemic inflammatory contribution to AF, whereas the higher SMD of 1.13 for periatrial EAT indicates a stronger localized effect, likely due to the atrial proximity.

Our finding of an association for periatrial EAT aligns with the critical role of left atrial (LA) structural remodeling in AF pathophysiology. The proximity of periatrial EAT to the atrial myocardium uniquely positions it to influence LA size and architecture—key determinants of AF substrate. Experimental and clinical data suggest that paracrine signaling from periatrial EAT (e.g., via inflammatory cytokines and profibrotic factors like transforming growth factor (TGF)-β and CTGF) may directly promote LA enlargement, fibrosis, and electrical remodeling, thereby creating a vulnerable substrate for AF initiation and maintenance [32, 34]. This is further supported by contemporary studies viewing EAT as part of an integrated “epicardial adipose complex” that interacts with myocardial fat infiltration and fibrosis to shape the arrhythmogenic substrate [34]. This potential indirect pathway—whereby periatrial EAT contributes to AF partly through LA dilation—could explain why its association appears more robust than that of total EAT in our analysis. Conversely, the more modest association observed for total EAT may reflect its role as a marker of generalized cardiometabolic risk and systemic inflammation, which influences AF through broader pathways less directly tied to LA structure.

However, despite the important role of EAT in the development of AF, EAT volume did not remain an independent predictor of AF recurrence in the study by Cruz et al. (2023) [35]. In this study, 350 consecutive patients after AF ablation were included. Univariable Cox regression showed that patients with an EAT volume ≥ 80 mL had an increased risk of AF recurrence (hazard ratio (HR), 1.65; 95% CI, 1.14–2.39; P = 0.007). However, after multivariable adjustment, EAT volume did not remain an independent predictor of AF recurrence (HR, 1.24; 95% CI, 0.83–1.87; P = 0.3) [35].

### Limitations

The limitations of our meta-analyses were that many of the included studies did not report important information, such as the type of AF or heart failure status. We acknowledge that the high heterogeneity of the results may be due to the differences in comorbidities (for example, heart failure), discrepancies in CT protocols, as well as variation in AF types. Secondly, differences between paroxysmal/persistent AF and EAT are not explored, though their pathologies may differ, so future studies are necessary.

### Conclusions

EAT presents a great interest in terms of cardiovascular remodeling and subsequent AF development. To date, it is well established that EAT is significantly associated with AF risk. This indicates the expedience of long-term Holter electrocardiogram (ECG) monitoring for patients with a large volume of EAT. In our meta-analysis periatrial EAT has a more convincing association with AF than total EAT. However, because of limited data about association between periatrial EAT and AF, further studies on this subject are necessary.

## Data Availability

The authors declare that data supporting the findings of this study are available within the article.
